# Optimization Modeling of Irreversible Carnot Engine from the Perspective of Combining Finite Speed and Finite Time Analysis

**DOI:** 10.3390/e23050504

**Published:** 2021-04-22

**Authors:** Monica Costea, Stoian Petrescu, Michel Feidt, Catalina Dobre, Bogdan Borcila

**Affiliations:** 1 Department of Engineering Thermodynamics, University POLITEHNICA of Bucharest, Splaiul Independentei 313, 060042 Bucharest, Romania; stoian.petrescu@yahoo.com (S.P.); catalina.dobre@upb.ro (C.D.); bbd1188@yahoo.com (B.B.); 2 Laboratory of Energetics, Theoretical and Applied Mechanics (LEMTA), URA CNRS 7563, University of Lorraine, 54518 Vandoeuvre-lès-Nancy, France; michel.feidt@univ-lorraine.fr

**Keywords:** irreversible Carnot engine, optimization, thermodynamics with finite speed, internal and external irreversibilities, entropy generation calculation, thermodynamics in finite time

## Abstract

An irreversible Carnot cycle engine operating as a closed system is modeled using the Direct Method and the First Law of Thermodynamics for processes with Finite Speed. Several models considering the effect on the engine performance of external and internal irreversibilities expressed as a function of the piston speed are presented. External irreversibilities are due to heat transfer at temperature gradient between the cycle and heat reservoirs, while internal ones are represented by pressure losses due to the finite speed of the piston and friction. Moreover, a method for optimizing the temperature of the cycle fluid with respect to the temperature of source and sink and the piston speed is provided. The optimization results predict distinct maximums for the thermal efficiency and power output, as well as different behavior of the entropy generation per cycle and per time. The results obtained in this optimization, which is based on piston speed, and the Curzon–Ahlborn optimization, which is based on time duration, are compared and are found to differ significantly. Correction have been proposed in order to include internal irreversibility in the externally irreversible Carnot cycle from Curzon–Ahlborn optimization, which would be equivalent to a unification attempt of the two optimization analyses.

## 1. Introduction

Recent work [1] has emphasized that an analysis using the *finite time of the process* rather convey to a “physical potential optimization” than to an “engineering optimization” of thermal machine [2]. What is called *physical optimization* could provide more realistic performance compared to reversible Carnot cycle one, but it is still overvalued with respect to the actual one. Thus, the results of the physical optimization can be considered as upper bounds for real machine performance [3,4,5].

Moreover, criticisms have been addressed [6,7,8,9,10,11] to the results of Finite Time Thermodynamics (FTT) analysis of thermal machines, claiming that it failed to keep the promises, at least from the engineer’s point of view. The main reason is the fact that FTT does not consider the internal losses generated by irreversibilities on a *fundamental basis*, since they have been introduced through a constant coefficient [12], factor of non-endoreversibility [13], degree of internal irreversibility [14], entropy variation ratio [15], ratio of two entropy differences [16], or entropy generation term as a function of temperature [17,18]. Therefore, the studies based on FTT approach cannot be effectively used by engineers for a better design and optimization study, leading to the conception and build of more efficient thermal machines since to apply optimization in a thermodynamic analysis, it needs to advance to the higher phases of the system design than the one based on endoreversibility assumption that is considered very early [10]. Furthermore, the internal irreversibilities contributed by the system components are inherently interconnected with external irreversibilities in real operation conditions, so the performance reported by FTT analysis may be even smaller compared to that of a real system [8].

These criticisms did not remain without reply [19,20,21,22,23]. Thus, some authors of the anti-criticism papers addressed the clarification of finite-time thermodynamics objectives and their inclusion in the efforts to approach the irreversible systems and their performance [21]. Others emphasized the meaning of time for thermodynamic processes, namely that of providing bounds by discussing nine general principles for finding bounds on the effectiveness of energy conversion [22] or bounds relative to the efficiency versus maximum power efficiency of heat engines [23].

However, regarding the usefulness of the FTT, the endoreversible model has the merit of launching nowadays the competition of finding new upper bounds of thermal machines performance, closer to the real one. Thus, progress has been made in the modeling and optimization of thermodynamic processes and cycles [24,25,26,27,28,29,30,31,32], with special attention to the common ones in thermal machines: Otto cycle [27], Stirling engine [28], Kalina cycle [30], and Brayton cycle [31,32]. The results obtained [30,31] have shown that besides the gains of FTT optimization with three or four objectives, the original results reported in the initial work of the FTT theory [3,4,5] are also revealed.

The engineering optimization is mainly concerned about internal irreversibility assessment by insight in dissipation mechanism, to approach and model the irreversible cycle performance. Both internal and external irreversibility are considered, conveying an actual optimization of thermal machine performance.

Although there is no operational Carnot machine, much has been written on the optimization of Carnot cycle, and in particular, on the heat engine cycle, endoreversible [33,34,35,36,37,38,39] or with internal and external irreversibilities [40,41,42,43,44,45,46,47,48,49,50,51,52,53,54,55,56,57,58,59,60,61]. One reason could be that the performance of the Carnot cycle represents upper bounds for actual operating machines. However, only in the 1990s was attention focused on analysis of the Carnot cycle that also includes internal irreversibilities [12,16,17,18,41,42,46,47,48,49].

The Thermodynamics with Finite Speed (TFS) has been shown to be able to provide analytical evaluation of internal irreversibilities in several machines (Stirling, Otto, Diesel, Brayton, Carnot) [60,61,62,63,64,65,66,67,68] and electrochemical devices [69], as a function of the speed of the piston. Actually, the finite speed of the piston (and process implicitly) is also responsible of external irreversibilities, namely the finite heat transfer rate from source to cycle fluid and then to sink. The computation scheme developed in TFS using the Direct Method is based on the *First Law of Thermodynamics for Processes with Finite Speed* that contains the main internal irreversibility causes of thermal machines expressed as a function of the average piston speed. By integration of the new expression of the First Law on each cycle process, analytical expression for performance (Power and Efficiency) is provided. It can be used to optimize theoretical cycles of actual thermal machines and most importantly, it was validated for 12 performing Stirling Engines (in 16 operational regimes) [63,64] and 4 Solar Stirling Motors [49,50].

In recent publications [54,55,56,57,58], it has been mentioned that only *Thermodynamics with Finite Speed* (TFS) developed the necessary tools to optimize thermal machines by considering internal losses in addition to external ones by analytical means. Based on these statements, it was concluded that using the above-mentioned achievements of TFS in combination with FTT tools could convey a more realistic and efficient approach of thermal machines.

The analytical approach relative to this combination is presented here by original models introducing irreversibilities step by step and leading to important results that are more accurate than those obtained by each irreversible thermodynamics branch separately.

Firstly, a brief presentation of the Curzon–Ahlborn modeling of an endoreversible Carnot engine is given, together with the discussion relative to the presence of the nice radical in other works.

Then, optimization models for a Carnot cycle engine in a closed system that operates with finite speed of the piston are presented. The speed is considered constant and equal to the average speed of the piston that moves with a classical rod–crankshaft mechanism; by using the First Law of Thermodynamics for Processes with Finite Speed and the Direct Method, the optimization analysis of this cycle with external and internal irreversibilities is developed. Heat losses between the two heat reservoirs temperature level through the engine are considered. External irreversibilities are due to the finite heat transfer rate at the source and sink are modeled by an irreversible coefficient added to the classical expression of heat transfer on isothermal process. Internal irreversibilities are included in the mathematical expression of the First Law of Thermodynamics for Processes with Finite Speed as non-dimensional pressure losses due to the non-uniformity of the fluid pressure in the cylinder and friction. The piston speed for maximum power and for maximum efficiency is found for a particular set of engine parameters and it is shown that the minimum entropy generation per cycle occurs at maximum power. This analysis provides lower values of Carnot cycle efficiency than predicted by the Curzon–Ahlborn approach that was considered for comparison.

A further development of the model aims to combine the analysis of the Carnot cycle engine with only external irreversibility from Finite Time Thermodynamics (FTT) with the main advantage of the Thermodynamics with Finite Speed (TFS) approach, namely the internal irreversibility quantification as a function of the speed of the process (piston). Thus, corrections of the power output, efficiency, and optimized cycle fluid temperature in FTT optimization results based on the calculated speed of processes from the duration time in FTT and average piston speed in TFS. It results that when internal ireversibilities (speeds and friction) are included, the performance predicted by a TFS analysis is better than that predicted by an FTT analysis.

The first unification attempt between TFS and FTT considers only pressure losses due to the non-uniformity of the pressure in the cylinder as a function of piston speed. The analytical development of the model provides modified Curzon–Ahlborn expression for the externally irreversible Carnot cycle to also include the internal irreversibility. Equations for the optimum cycle temperature, maximum power, and efficiency for the internally and externally irreversible cycle are presented. The corrections are shown to increase with increased piston speed and to be significant at high but realizable piston speeds. The optimum temperature corresponding to maximum power is shown to increase with increased piston speed.

Then, a further step in the unification attempt between TFS and FTT is done by considering in addition to the Finite Speed, two other causes of internal irreversibility given by friction and throttling. Thus, based on the first unification achievement, new expressions are derived for the power output and efficiency of the direct Carnot cycle with finite speed processes. The results emphasize optimum speed values generating maximum power output, as well as the effect of irreversibilities on the optimum high temperature of the cycle. 

The overview on the results of these models emphasizes that a significant difference exists between the results of the two optimization analyses in the sense that FTT optimization seems to be an upper bound when compared to the engineering optimization based on TFS and the Direct Method.

## 2. Optimization Models of Carnot Cycle Engine

### 2.1. Models in Thermodynamics in Finite Time Analysis Seeking for Maximum Power Output of Carnot Cycle Engine

The Curzon–Ahlborn modeling of the Carnot-type engine [3] refers to a cycle that is internally reversible but with no thermal equilibrium between the working fluid and the thermal reservoirs during the isothermal heat input and heat rejection, respectively. Furthermore, there exists a finite time duration of heat transfer given by Newton’s heat transfer law during the isothermal processes. The expression of the power output of the Curzon and Ahlborn cycle allows a maximum for which the corresponding efficiency is given by what was called nice radical.

Actually, the efficiency of a Carnot engine is treated for the case where the power output is limited by the rates of heat transfer to and from the working substance. It is shown that the efficiency, *η_CA_*, at maximum power output is given by the expression *η_CA_* = 1 − (*T*_2_/*T*_1_)^1/2^ where *T*_1_ and *T*_2_ are the respective temperatures of the heat source and heat sink. It results in an efficiency less than the one introduced by Carnot (*η* = 1 − (*T*_2_/*T*_1_)), and it is shown that the existing engines performance is well described by the above result.

Before the Curzon and Ahlborn analysis, a similar approach aiming to maximize the power output and the nice radical has appeared in Chambadal modeling of the Carnot engine [4], but its model used heat capacity rate instead of heat conductances.

Almost at the same time, Novikov [5] has also found the nice radical.

The above-mentioned models and mainly the Curzon–Ahlborn one, which remain as references for the Carnot machine optimization in the frame of what was called Thermodynamics in Finite Time.

### 2.2. Models of Irreversible Carnot Cycle Engine in Thermodynamics with Finite Speed

#### 2.2.1. First Law of Thermodynamics for Processes with Finite Speed in Closed System

The optimization modeling presented in this section proceeds from a basis of thermodynamic fundamentals, systematically detailed and developed, starting from a unique equation called the *First Law of Thermodynamics for Processes with Finite Speed* [59,70,71,72,73,74,75,76,77,78,79]. The advantages of using this equation instead of the one from Classical Reversible Thermodynamics consists of its capability to account for both causes and mechanisms of irreversibility generation in complex cycles or real machines such as Stirling Engines, as well as in other cycles such as Otto, Diesel, Brayton, and Carnot cycles [60,71,72,73]. In addition, it is capable to consider both internal and external irreversibilities.

By integrating this equation for irreversible process step by step on each transformation of the cycle, the efficiency and power output are determined *analytically*. These expressions contain the *causes of irreversibility*, namely, the *finite speed of the piston*, an important parameter that can be optimized, for *Maximum Efficiency* or *Maximum Power*.

The mathematical expression of the First Law of Thermodynamics for Processes with Finite Speed in a closed system in its differential form is [59,70,71,72,73,74,75,76,78]:

(1) $$dU=\delta Q-p_{av,i}\left(1\pm \frac{aw}{c}\pm \frac{f\cdot \Delta p_{f}}{p_{av,i}}\right)\;dV,$$

and the irreversible work for these processes [59,70,71,72,73,74,75,76,78]:

(2) $$\delta W_{irrev}=p_{av,i}\left(1\pm \frac{aw}{c}\pm \frac{\Delta p_{f}}{p_{av,i}}\right)\;dV$$

where *U*—internal energy, *Q*—heat, *W*—mechanical work, *p_av,i_*—instantaneous average pressure of the gas, *w*—average speed of the piston, *c*—average molecular speed, Δ*p_f_*—pressure losses due to friction, *a*—coefficient depending the gas nature, *f*—coefficient relative to the amount of heat generated by friction that remains in the cycle, and *V*—volume.

In the previous equations, the plus sign corresponds to the compression processes and the minus sign corresponds to the expansion ones.

Regarding the terms appearing in the right member, the first term in the parenthesis accounts for the irreversibility generated by the Finite Speed of the piston, *w*, and due to the non-uniformity of the pressure in the cylinder. Therefore, the pressure on the piston *p_p_* is larger during compression and smaller during expansion than the pressure on the head of the cylinder *p_c_*, and this is also the case for the instantaneous average pressure in the gas *p_av.i_* [47,59,60,61,76]. The experimental verification of this term is described in references [51,59,60,61]. The second term in the parenthesis takes into account the irreversibility generated by the friction between moving parts of the machine (piston–cylinder, bearings, etc.) [47,60,61]. When the processes in the machine involve internal throttling, a third term is added in the First Law for Processes with Finite Speed [47,60,61], playing an important role in the optimization of Stirling machines [51,59,60,61,62,63,64,65,66,67,77,80]. This term is less important in the Carnot cycle modeling, so that it is neglected in this study.

Other terms from the right member of Equations (1) and (2) have the following expressions:

(3) $$a=\sqrt{3\gamma },\;c=\sqrt{3RT},$$

with *γ*—ratio of specific heat at constant pressure and constant volume, and *R*—gas specific constant.

The pressure losses due to friction expressed as function of rotation per minute and based on their experimental evaluation for classical thermal engines operating upon Otto and Diesel cycles [81] were adapted to speed [76], and their expression resulted as:

(4) $$\Delta p_{f}=\left(0.97+0.045w\right)/N$$

where *N*—parameter depending on structural characteristics of the engine.

Note that Equations (1) and (2) completed by Equations (3) and (4) clearly show that the finite speed of the piston is responsible for all irreversibility causes, since it appears in both terms in the parentheses.

#### 2.2.2. Model of Carnot Cycle Engine with Analytically Modeled Internal and External Irreversibility

**Figure 1:**
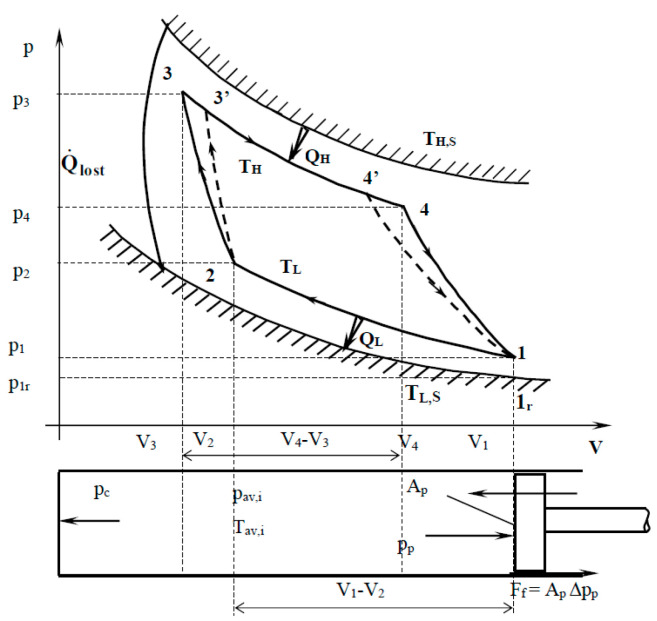
Carnot engine cycle with finite speed of the piston illustrated in p-V diagram [48,49].

The cyclic system of a Carnot heat engine, including irreversibilities of finite-rate heat transfer between the gas in the thermal engine and its heat reservoirs, heat leakage between the reservoirs, and internal dissipations of the working fluid, is shown schematically in Figure 1 [48,49]. The working fluid in the system is alternately connected to a hot reservoir at constant temperature *T_H,S_* and to a cold reservoir at constant temperature *T_L,S_* and its temperatures are, respectively, *T_H_* and *T_L_*.

Heat losses between the two heat reservoirs temperature level through the engine are considered by the heat rate term ${\dot{Q}}_{lost}$. In addition, irreversible adiabatic processes are shown by the curves 2-3′ and 4′-1.

Inside the cylinder with the piston illustrated in the bottom side of Figure 1 appears several pressures that are used in a process with finite speed analysis: on the piston, *p_p_*, on the cylinder, *p_c_*, and the instantaneous average pressure in the gas, *p_av,i_*.

By integrating Equations (1) and (2) over the isothermal processes of the Carnot cycle, the following expressions for the energy exchanges are dependent of the average piston speed yield:

- The irreversible heat received by the cycle gas from the source:

(5) $$Q_{H}=z_{H}^{\prime }\cdot mRT_{H}ln\;\frac{V_{4}}{V_{3}}=z_{H}^{\prime }\cdot mRT_{H}\cdot ln\varepsilon ,$$

with $z_{H}^{\prime }$—irreversible coefficient that accounts for a limited heat input in the cycle due to the finite speed of the process:

(6) $$z_{H}^{\prime }=\left(1-\frac{aw}{\sqrt{3RT_{H}}}-\frac{f\cdot \Delta p_{f}}{p_{av,34}}\right).$$

This irreversible coefficient shows that regardless of the heat available at the source, the cycle gas can only receive a limited amount of heat from the source.

- The irreversible heat rejected by the cycle gas to the sink:

(7) $$Q_{L}=z_{L}^{\prime }\cdot mRT_{L}ln\;\frac{V_{2}}{V_{1}}=-z_{L}^{\prime }\cdot mRT_{L}\cdot ln\varepsilon ,$$

with $z_{L}^{\prime }$—irreversible coefficient that accounts for a limited heat rejected by the cycle gas to the sink due to the finite speed of the process:

(8) $$z_{L}^{\prime }=\left(1+\frac{aw}{\sqrt{3RT_{L}}}+\frac{f\cdot \Delta p_{f}}{p_{av,12}}\right).$$

- The irreversible work produced/consumed during the isothermal processes of the cycle:

(9) $$W_{H,w}=z_{H}\cdot mRT_{H}\cdot ln\varepsilon ,$$

(10) $$\left|W_{L,w}\right|=z_{L}\cdot mRT_{L}\cdot ln\varepsilon ,$$

with the corresponding irreversible coefficients:

(11) $$z_{H}=\left(1-\frac{aw}{\sqrt{3RT_{H}}}-\frac{\Delta p_{f}}{p_{av,34}}\right),$$

(12) $$z_{L}=\left(1+\frac{aw}{\sqrt{3RT_{L}}}+\frac{\Delta p_{f}}{p_{av,12}}\right).$$

with

(13) $$mR=P_{1r}V_{1r}/T_{1r},$$

and

(14) $$T_{1r}=T_{L,S},\;V_{1r}=V_{1}.$$

and

(15) $$\frac{V_{4}}{V_{3}}=\frac{V_{1}}{V_{2}}=\varepsilon .$$

The work per cycle results from Equations (9) and (10) as:

(16) $$W_{cycle,w}=mR\left(z_{H}T_{H}-z_{L}T_{L}\right)ln\varepsilon .$$

**Figure 2:**
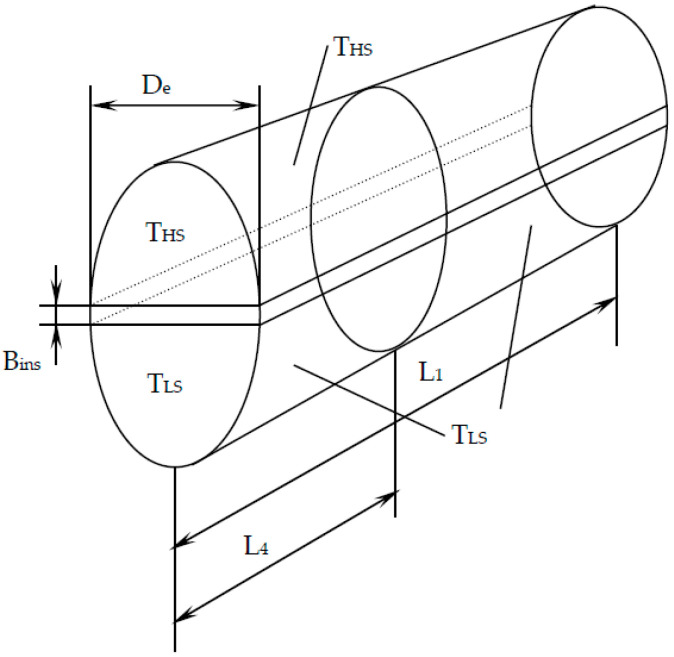
The cylinder configuration used in heat transfer area computation [48,49].

The non adiabaticity of the engine suggested in Figure 1 by the term ${\dot{Q}}_{lost}$ is better explained in Figure 2 by the insulating wall between the two semi-cylinders that form the heat conduction path between the heat source and sink.

The heat transfer rate lost through this conduction path is:

(17) $${\dot{Q}}_{lost}=k_{ins}A_{lost}\left(T_{HS}-T_{LS}\right)/B_{ins},$$

where *k_ins_*—thermal conductivity of the insulation, and *B_ins_*—insulation thickness.

Equation (17) expressed on the cycle becomes:

(18) $$Q_{lost,cycle}={\dot{Q}}_{lost}\cdot \tau_{cycle}.$$

The cycle time duration can be expressed as:

(19) $$\tau_{cycle}=\frac{2\left(V_{1}-V_{3}\right)}{wA_{p}},$$

with *A_p_*—piston area.

The area associated to the heat transfer rate lost between the source and sink yields (see Figure 2):

(20) $$A_{lost}=\left(D+2L_{4}\right)\left(D_{e}-D\right),$$

where *D* is the inner diameter of the cylinder.

This heat transfer rate lost per cycle will modify the heat supply from the source and the heat rejected to the sink as follows:

(21) $$Q_{H,tot}=Q_{H}+Q_{lost,cycle},$$

(22) $$\left|Q_{L,tot}\right|=\left|Q_{L}\right|+Q_{lost,cycle}.$$

In the above equations, the heat input to the cycle gas and heat rejected from the gas to the sink may be considered those already given by Equations (5) and (7), or it can be expressed in terms of heat transfer as follows:

(23) $$Q_{H}=U_{H}\left(w\right)\cdot A_{H}\cdot \left(T_{H,S}-T_{H}\right)\cdot \tau_{H},$$

(24) $$\left|Q_{L}\right|=U_{L}\left(w\right)\cdot A_{L}\cdot \left(T_{L}-T_{L,S}\right)\cdot \tau_{L}.$$

where *U_H_*(*w*) and *U_L_*(*w*) are the overall heat transfer coefficient during the heat exchange at the source and sink, respectively, and *A_H_* and *A_L_* are the area of the heat transfer surfaces. 

The heat transfer expressed using the Finite Speed analysis (Equations (5) and (7)) should be the same as the heat transfer corresponding to the above Equations (23) and (24). Therefore, the two equalities allow expressing the *temperature of the gas at the hot end and at the cold end* respectively, in connection with the source and sink temperature:

(25) $$T_{H}=T_{H,S}\cdot {\left[1+\frac{z_{H}^{\prime }\cdot mR\cdot ln\varepsilon }{U_{H}\left(w\right)\cdot A_{H}\cdot \tau_{H}}\right]}^{-1}$$

(26) $$T_{L}=T_{L,S}\cdot {\left[1-\frac{z_{L}^{\prime }\cdot mR\cdot ln\varepsilon }{U_{L}\left(w\right)\cdot A_{L}\cdot \tau_{L}}\right]}^{-1}.$$

The overall heat transfer coefficients of the heat exchanger at source and sink, *U_L_*, *U_H_* are calculated based on average bulk fluid temperatures by using well-known equations [82]:

(27) $$Nu_{D}=\{\begin{array}{ll} 1.86{\left(Re_{D}Pr\right)}^{\frac{1}{3}}{\left(\frac{D}{L}\right)}^{\frac{1}{3}}{\left(\frac{\mu }{\mu_{wall}}\right)}^{0.14}, & for\;{Re}_{D}\;\leq \;2300\\ 0.023\;{Re}_{D}^{0.8}{Pr}^{n}, & for\;{Re}_{D}\;\geq \;3000 \end{array},$$

with *n* = 0.4 for heating, respectively, *n* = 0.3 for cooling.

Similarly, the dynamic viscosity and the thermal conductivity of the gas are calculated using polynomial functions [64], based on the bulk gas temperature.

The contact time per cycle for the heat transfer from the heat source to the engine corresponding to the isothermal process is:

(28) $$\tau_{H}=\left(L_{4}-L_{3}\right)/w=\frac{L_{1}\left(1-\frac{1}{\varepsilon }\right){\left(\frac{T_{L}}{T_{H}}\right)}^{\frac{1}{\gamma }-1}}{w},$$

while the contact time per cycle for heat transfer from the gas engine to the sink is:

(29) $$\tau_{L}=\left(L_{1}-L_{2}\right)/w=\frac{L_{1}\left(1-\frac{1}{\varepsilon }\right)}{w}.$$

The area for the heat transfer between the source and the hot gas during the isothermal heat addition process (see Figure 2) is:

(30) $$A_{H}=0.5D\left(\frac{\pi D}{4}-B_{ins}\right)+0.5L_{1}\left(1+\frac{1}{\varepsilon }\right)\left(\frac{\pi D}{2}-B_{ins}\right)\cdot {\left(\frac{T_{L}}{T_{H\;}}\right)}^{\frac{1}{\gamma }-1}.$$

Similarly, the area for heat transfer between the cold gas and the sink during the isothermal heat rejection process is expressed as:

(31) $$A_{L}=0.5D\left(\frac{\pi D}{4}-B_{ins}\right)+0.5L_{1}\left(1+\frac{1}{\varepsilon }\right)\left(\frac{\pi D}{2}-B_{ins}\right),$$

with

(32) $$\frac{L_{1}}{\varepsilon }=L_{2}.$$

The power output of the irreversible Carnot engine is given by:

(33) $$P_{\Delta T,w,Q_{lost}}=\frac{W_{cycle,w}}{\tau_{cycle}}.$$

The efficiency of the Carnot cycle with internal and external irreversibility is:

(34) $$\eta_{\Delta T,w,Q_{lost}}=1-\frac{\left|Q_{L,w}\right|}{Q_{H,w}}=1-\frac{T_{L}}{T_{H}}\cdot \frac{z_{L}^{\prime }}{z_{H}^{\prime }}.$$

Then, the entropy generation per cycle can be expressed as:

(35) $$\Delta S_{cycle}=\frac{Q_{H,w}}{T_{H}}+\frac{Q_{L,w}}{T_{L}}=mRln\varepsilon \cdot \left(z_{H}^{\prime }-z_{L}^{\prime }\right),$$

and its corresponding expression per unit time is:

(36) $${\dot{S}}_{gen}=\frac{\Delta S_{cycle}}{\tau_{cycle}}.$$

The results of this optimization model will be given in Section 3.

### 2.3. The Curzon–Ahlborn Model of the Carnot Cycle Engine Combined with the Analysis Based on Thermodynamics with Finite Speed (TFS)

The model aims to combine the analysis of the Carnot cycle engine with only external irreversibility in Thermodynamics in Finite Time (FTT) with the main advantage of the Thermodynamics with Finite Speed (TFS) approach, namely the internal irreversibility quantification as a function of the speed of the process.

The main differences of this model compared to the previous one are represented by:

- The absence of heat losses *Q_lost_*, in order to consider similar cycles in both analyses.
- The presence of losses in the work expression, so that the work lost in the two adiabatic processes due to finite speed is obtained by integrating the irreversible work for processes with finite speed in the processes 2-3′ and 4′-1 (Equation (2)) and subtracting the reversible work in the processes 2-3 and 4-1 (see Figure 1):

(37) $$W_{lost,\;ad,\;int}=\left(\frac{aw}{c_{23^{\prime }}}+\frac{\Delta p_{f}}{p_{23^{\prime }}}\right){\left(V_{3^{\prime }}-V_{2}\right)}_{23^{\prime }}-\left(\frac{aw}{c_{4^{\prime }1}}+\frac{\Delta p_{f}}{p_{4^{\prime }1}}\right){\left(V_{1}-V_{4^{\prime }}\right)}_{4^{\prime }1}.$$

where *p*_23′_ and *p*_4′1_ are the average gas pressure on the irreversible adiabatic compression and expansion, respectively.

This lost work term is then subtracted from the work per cycle given by Equation (16), since it does not include the effect of internal irreversibilities of the adiabatic processes. 

By including this lost work term in the analysis, an expression for the efficiency of the Carnot cycle, considering all internal and external irreversibilities yields as:

(38) $$\eta_{\Delta T,w,f}=\left(\frac{z_{H}}{z_{H}^{\prime }}-\frac{z_{L}\cdot T_{L}}{z_{H}^{\prime }\cdot T_{H}}\right)-I_{ad}\frac{1-T_{L}/T_{H}}{z_{H}^{\prime }\left(\gamma -1\right)ln\varepsilon },$$

where the irreversible adiabatic process contribution of the internal irreversibility of the cycle, due to the finite piston speed and friction, *I_ad_*, results as:

(39) $$I_{ad}=aw\left(\frac{1}{c_{23^{\prime }}}+\frac{1}{c_{4^{\prime }1}}\right)+\Delta p_{f}\left(\frac{1}{p_{23^{\prime }}}+\frac{1}{p_{4^{\prime }1}}\right).$$

Note that the second term in Equation (38) is obtained by integration of the First Law for Processes with Finite Speed (TFS) for the adiabatic processes 23′ and 4′1 (see Figure 1), Equations (1) and (2).

The combination of the two analyses based on FTT and TFS models will include a similar term to that given by Equation (39) in the Curzon–Ahlborn approach. As previously mentioned, this approach included the time duration of the cycle processes, with the assumption that the adiabatic processes occur rapidly and accordingly consume far less time than the isothermal processes. Based on this assumption, the FTT and TFS analyses can be rationally compared only if the Carnot cycle engine dimensions and number of cycles per unit time are made equal in both cases. In a TFS analysis, the speed of the piston, *w*, is assumed constant in each of the four processes and equals the average speed based on the number of cycles per unit time. However, in a Curzon–Ahlborn type analysis (FTT optimization), the speed of isothermal compression *w_L_*, the speed of isothermal expansion *w_H_*, and the speed of the adiabatic processes *w_ad_* (assumed equal for both adiabatic processes), are calculated. The result must be consistent with the total cycle time optimized for maximum power.

When this comparison is performed, the following process speeds, in terms of the average speed, are obtained (see Figure 2) [49]:

(40) $$w_{L}=\frac{a^{\prime }\left(L_{1}-L_{2}\right)\left(1+Z^{*}\right)}{2L_{1}/w},$$

(41) $$w_{H}=\frac{a^{\prime }\left(L_{4}-L_{3}\right)\left(1/Z^{*}+1\right)}{2L_{1}/w},$$

(42) $$w_{ad}=\frac{a^{\prime }w\left[\left(L_{2}-L_{3}\right)+\left(L_{1}-L_{4}\right)\right]}{2L_{1}\left(a^{\prime }-1\right)},$$

where Z*—ratio of the optimized duration of the isothermal processes in the Curzon–Ahlborn treatment (FTT), *a*’—coefficient depending on time to speed transfer.

The optimized temperatures in the Curzon–Ahlborn analysis [3] are expressed based on corresponding optimized times for each process, as follows:

(43) $$T_{L,FTT}=T_{L}\frac{1+\sqrt{\frac{T_{H}}{T_{L}}}\cdot \frac{1}{Z^{*}}}{1+\frac{1}{Z^{*}}},$$

(44) $$T_{H,FTT}=T_{H}\frac{1+\sqrt{\frac{T_{L}}{T_{H}}}\cdot Z^{*}}{1+Z^{*}}.$$

By using the above expressions of temperatures and including the effect of internal irreversibility, the corresponding power of Carnot cycle in FTT analysis is:

(45) $$Power_{FTT}=\frac{\frac{A_{L}U_{L}}{a^{\prime }}.{\left(\sqrt{T_{H}}-\sqrt{T_{L}}\right)}^{2}}{{\left(Z^{*}+1\right)}^{2}}-\left(W_{loss,ad,int}+W_{loss,isot,int}\right)\frac{1}{\tau_{cycle}}.$$

Equation (45) appears as a combination of the two analyses as the first term is the original Curzon–Ahlborn term [3] taking account of only external irreversibilities generated by the temperature difference, and the second term accounts for internal irreversibilities generated by the finite speed and friction from the TFS approach.

Nevertheless, a simpler expression of the power output can be also given as:

(46) $$Power_{\Delta T,w,f,FTT}=Q_{H}\cdot \eta_{\Delta T,w,f,FTT}^{\prime }\cdot \frac{1}{\tau_{cycle}},$$

where the efficiency term contains all irreversibility causes of the Carnot cycle engine.

The passage from the efficiency of the Carnot cycle including only external irreversibilities and corresponding to maximum power output in the original Curzon–Ahlborn analysis [3]:

(47) $$\eta_{\Delta T,FTT}=1-\frac{T_{L,FTT}}{T_{H,FTT}}=1-\sqrt{\frac{T_{LS}}{T_{HS}}},$$

will be performed here by including the effects of internal irreversibilities. Similarly, Equations (5)–(12) are expressed by evaluating Z_FTT_ and Z′_FTT_ irreversible coefficients at the appropriate speeds (*w_L_* and *w_H_*) on the isothermal processes at *T_L_* and *T_H_* respectively, and on the adiabatic processes (*w_ad_*) conveying to the following corrected efficiency:

(48) $$\eta_{irr,int,FTT}=\frac{Z_{H,FTT}}{Z_{H,FTT}^{\prime }}-\frac{Z_{L,FTT}\cdot T_{L,FTT}}{Z_{L,FTT}^{\prime }\cdot T_{H,FTT}}-I_{ad}^{\prime }\frac{1-T_{L,FTT}/\cdot T_{H,FTT}}{Z_{H,FTT}^{\prime }\left(\gamma -1\right)ln\varepsilon }$$

where the equivalent term *I_ad_*′ to that from Equation (39) is similar, but it is based on *w_ad_* (Equation (42)) instead of *w* and also on the resulting temperatures and pressures from the Curzon–Alhborn. Ref. [3] analysis of the Carnot cycle completed by TFS tools (Equations (43) and (44)).

### 2.4. Unification Attempts of Thermodynamics in Finite Time and Thermodynamics with Finite Speed Analyses

The first unification attempt is based on [47] that had a very important role in the development of Thermodynamics with Finite Speed (TFS) and the Direct Method, for analytical evaluation of the performances of irreversible cycles with internal and external irreversibilities. Later, it was completed by [31,34].

**Figure 3:**
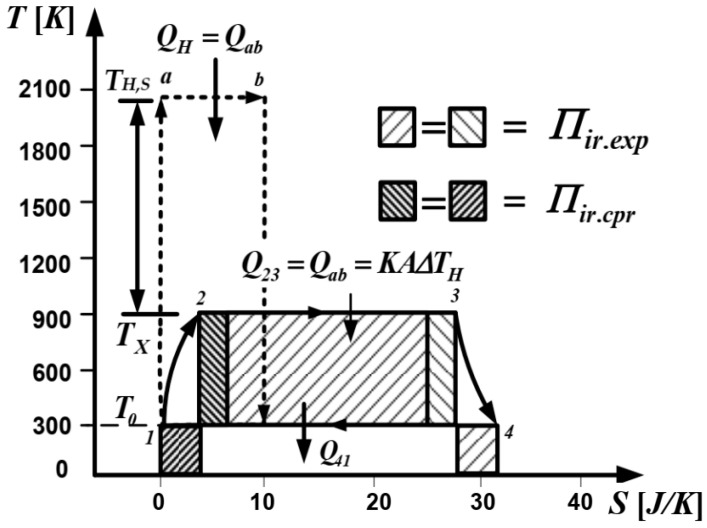
Carnot engine cycle with internal irreversibilities illustrated in T-S diagram [47,52].

Specific issues addressed in this model are illustrated on cycle Carnot engine represented in *T-S* coordinates in Figure 3. There are shown to have external irreversibility due to heat transfer from the source (with fixed temperature *T_H,S_*) to the cycle temperature at the hot end, *T_X_*, during the isothermal heat addition process 2–3. Then, internal irreversibilities due to the finite piston speed are considered during only the adiabatic compression and expansion processes. The sink temperature and the cycle temperature at the cold end are the same. The sink temperature, *T*_0_, is fixed, while the cycle temperature at the hot end, *T_X_*, is a variable.

Another novelty compared to previous model consists of the use of entropy variation calculation on the irreversible cycle processes that will provide a term in the cycle efficiency expression that could unify the two analyses.

The first unification attempt is based on the First Law of Thermodynamics for Processes with Finite Speed [70,71,72,73] in its reduced form that considers only the internal irreversibility due to the finite speed of the piston:

(49) $$dU=\delta Q-p_{av,i}\left(1\pm \frac{aw}{c}\right)\;dV.$$

From the equation for adiabatic irreversible processes of ideal gases with constant specific heats that is derived from Equation (49) by integration [72,73,75,76], one can express the temperature *T*_2_ at the end of an irreversible adiabatic process as

(50) $$T_{2}=\frac{{\left(1\pm \frac{aw}{c_{1}}\right)}^{2}}{{\left(1\pm \frac{aw}{c_{2}}\right)}^{2}}T_{1}{\left(\frac{V_{1}}{V_{2}}\right)}^{\gamma -1}=\delta_{irr}T_{1}{\left(\frac{V_{1}}{V_{2}}\right)}^{\gamma -1},$$

where *γ* is the ratio of the specific heat at constant pressure and at constant volume.

For a compression process with finite speed *w* << *c*, one could express $\delta_{irr.cpr}$ as follows:

(51) $$\delta_{irr,cpr}=\frac{{\left(1+\frac{aw}{c_{1}}\right)}^{2}}{{\left(1+\frac{aw}{c_{2}}\right)}^{2}}≅{\left[\left(1+\frac{aw}{c_{1}}\right)\left(1-\frac{aw}{c_{2}}\right)\right]}^{2}={\left[1+\frac{aw}{c_{1}}-\frac{aw}{c_{2}}\right]}^{2},$$

if a^2^*w^2^* << *c*_1_·*c*_2_ and the corresponding term is neglected.

Note that for compression, the plus sign is used in parenthesis.

Note that the average molecular speed *c*_2_ depends on temperature *T*_2_ that contains $\delta_{irr.cpr}$. Thus, the calculation should be done by using approximations.

The first approximation considers the temperature at the end of the reversible adiabatic compression for which one gets (see Equation (3)):

(52) $$T_{2}=T_{1}{\left(\frac{V_{1}}{V_{2}}\right)}^{\gamma -1}\;\Rightarrow \;c_{2}=c_{1}{\left(\frac{V_{1}}{V_{2}}\right)}^{\frac{\gamma -1}{2}}.$$

By substituting Equation (52) in Equation (51), a first evaluation of $\delta_{irr.cpr}$ is done:

(53) $$\delta_{irr.cpr}={\left[1+\frac{aw}{c_{1}}-\frac{aw}{c_{1}}{\left(\frac{V_{2}}{V_{1}}\right)}^{\frac{\gamma -1}{2}}\right]}^{2}.$$

Note that a more precise approximation is possible by combining Equations (50) and (53) that yields:

(54) $$T_{2}=\delta_{irr.cpr}T_{1}{\left(\frac{V_{1}}{V_{2}}\right)}^{\gamma -1},$$

and a better approximation for the adiabatic irreversible coefficient is given by:

(55) $$\delta_{irr.cpr}^{\prime }={\left[1+\frac{aw}{c_{1}}-\frac{aw}{c_{1}}{\left(\frac{V_{2}}{V_{1}}\right)}^{\frac{\gamma -1}{2}}{\left(\delta_{irr,cpr}\right)}^{-\frac{1}{2}}\right]}^{2}.$$

For simplicity, the first approximation expression of the adiabatic irreversible coefficient (Equation (53)) is used hereafter.

The entropy variation computation in the case of an adiabatic irreversible process of compression with finite speed when the results from Equations (50) and (53) are introduced in the classical formula of Δ*S*:

(56) $$\Delta S=S_{f}-S_{i}=mc_{v}ln\frac{T_{f}}{T_{i}}+mRln\frac{V_{f}}{V_{i}},$$

which provides:

(57) $$\Delta S_{irr,cpr}=mc_{v}ln{\left[1+\frac{aw}{c_{1}}-\frac{aw}{c_{1}}{\left(\frac{V_{2}}{V_{1}}\right)}^{\frac{\gamma -1}{2}}\right]}^{2}.$$

Similarly, the entropy variation expression on the adiabatic irreversible expansion can be derived showing that the only difference consists in the change of signs in the parentheses, so that one can give a general form of both compression and expansion processes, as:

(58) $$\Delta S_{ad,⥂irr}^{w}=mc_{v}ln{\left[1\pm \frac{aw}{c_{1}}\mp \frac{aw}{c_{1}}{\left(\frac{V_{2}}{V_{1}}\right)}^{\frac{\gamma -1}{2}}\right]}^{2}.$$

By using Equations (56) and (58) in the present analysis on the two irreversible adiabatic processes and on the isothermal expansion, the following expressions result:

(59) $$\Delta S_{ad.irr.cpr}^{w}=\Delta S_{12}=mc_{v}ln\left(\alpha_{1}\right),\;\mathrm{with}\;\alpha_{1}={\left[1+\frac{aw_{cpr}}{c_{1}}-\frac{aw_{cpr}}{c_{1}}{\left(\frac{V_{2}}{V_{1}}\right)}^{\frac{\gamma -1}{2}}\right]}^{2},$$

(60) $$\Delta S_{ad.irr.exp}^{w}=\Delta S_{34}=mc_{v}ln\left(\alpha_{2}\right),\;\mathrm{with}\;\alpha_{2}={\left[1-\frac{aw_{exp}}{c_{3}}+\frac{aw_{exp}}{c_{3}}{\left(\frac{V_{4}}{V_{3}}\right)}^{\frac{\gamma -1}{2}}\right]}^{2},$$

(61) $$\Delta S_{23}=S_{3}-S_{2}=mRln\frac{p_{2}}{p_{3}}.$$

with *c_v_*—specific heat at constant volume, *R*—specific constant of the cycle fluid.

Then, the actual thermal efficiency of the Carnot cycle engine with irreversibilities can be expressed based on previous calculation (see Figure 3) as:

(62) $$\eta_{act}=1-\frac{Q_{41}}{Q_{23}}=1-\frac{T_{C}\Delta S_{14}}{T_{X}\Delta S_{23}}=1-\frac{T_{0}\left(\Delta S_{23}+\Delta S_{12}+\Delta S_{34}\right)}{T_{X}\Delta S_{23}},$$

and together with Equations (59)–(61), the following expression results:

(63) $$\eta_{act}=1-\frac{T_{0}}{T_{X}}\left[1+\frac{2ln\left(\alpha_{1}\alpha_{2}\right)}{\left(\gamma -1\right)ln\frac{p_{2}}{p_{3}}}\right].$$

When the piston speed is much less than the average molecular speed, namely *aw_cpr_* << *c_1_*, and *a_exp_* << *c_3_*, one gets a simplified form of Equation (63):

(64) $$\eta_{act}=1-\frac{T_{0}}{T_{X}}\left[1+\frac{2\left(\beta_{1}+\beta_{2}\right)}{\left(\gamma -1\right)ln\frac{p_{2}}{p_{3}}}\right],$$

where

(65) $$\beta_{1}=\frac{aw_{cpr}}{c_{1}}\left(1-\sqrt{\frac{T_{0}}{T_{X}}}\right),$$

(66) $$\beta_{2}=\frac{aw_{exp}}{c_{3}}\left(\sqrt{\frac{T_{X}}{T_{0}}}-1\right).$$

For the same speed of the piston on the two adiabatic processes of the cycle, Equation (64) becomes:

(67) $$\eta_{act}=1-\frac{T_{0}}{T_{X}}\left\{1+\frac{4aw}{c_{1}}\frac{\left(1-\sqrt{\frac{T_{0}}{T_{X}}}\right)}{\left(\gamma -1\right)ln\frac{p_{2}}{p_{3}}}\right\}.$$

Once having the actual efficiency of the cycle, the power output of the engine can be easily derived as:

(68) $${\dot{W}}_{act}={\dot{Q}}_{H}\eta_{act}=U_{H}A_{H}\left(T_{H,S}-T_{X}\right)\eta_{act}.$$

To render the model more general, a non-dimensional form of the power output of the Carnot engine will be optimized, namely:

(69) $$P_{ND}=\frac{{\dot{W}}_{act}}{U_{H}A_{H}T_{H,S}}.$$

Moreover, the actual efficiency is expressed as a product of the Carnot reversible efficiency:

(70) $$\eta_{CC}=\left(1-\frac{T_{0}}{T_{X}}\right),$$

and the second law efficiency accounting for irreversibilities:

(71) $$\eta_{IIad.irr}^{w}=\left[1-\frac{C\left(\frac{T_{0}}{T_{X}}\right)}{\left(1+\sqrt{\frac{T_{0}}{T_{X}}}\right)}\right],$$

with the internal irreversible coefficient *C* given by:

(72) $$C=\frac{4aw}{c_{1}\left(\gamma -1\right)ln\frac{p_{2}}{p_{3}}}.$$

By combining Equation (69) with Equations (68), (70)–(72) and term rearrangement, one gets:

(73) $$P_{ND}=\left(1-\frac{T_{X}}{T_{H,S}}\right)\left(1-\frac{T_{0}}{T_{X}\Phi }\right),$$

With

(74) $$\Phi =\frac{1}{1+C\left(1-\sqrt{\frac{T_{0}}{T_{X}}}\right)}.$$

Note that for a given cycle fluid, coefficient Φ depends only on the fluid temperature at the hot end, *T_X_*, and the piston speed, *w*. Thus, the non-dimensional power (Equation (73)) is seen to be a complex function of *T_X_* and the piston speed by the term *C*. Searching for an analytic expression of the optimum temperature to maximize the non-dimensional power can be done in the first approximation, for Φ = constant in Equation (73). This is in good agreement with Ibrahim’s approach [16], where for Φ constant, the expression of the optimal temperature of the cycle fluid at the hot end that maximizes the power output of the engine was established as:

(75) $$T_{X}^{\max P_{ND}}\to T_{opt}=\sqrt{\frac{T_{H,S}\cdot T_{0}}{\Phi }}.$$

Although this is a simple expression, the value of Φ is not known. It is indicated as a parameter with a given (not computed) value.

In the present analysis, one can approximate the value of *T_opt_* by iterations. Thus:

- For *w =* 0, which means an internally reversible cycle, Equations (72) and (74) lead to Φ = 1, so that Equation (75) becomes:

(76) $$T_{opt}^{\left(w=0\right)}=\sqrt{T_{H,S}\cdot T_{0}}.$$

- For *w* ≠ 0, by combining Equations (74) and (76), a first approximation of the term responsible for cycle irreversibilities is expressed as:

(77) $$\Phi_{w}={\left[1+C\left(1-\sqrt[{4}]{\frac{T_{0}}{T_{H,S}}}\right)\right]}^{-1},$$

and the corresponding optimum temperature yields from Equation (75) as:

(78) $$T_{opt}^{\left(w\neq 0\right)}=\sqrt{T_{H,S}\cdot T_{0}\left[1+C\left(1-\sqrt[{4}]{\frac{T_{0}}{T_{H,S}}}\right)\right]}.$$

Equation (78) is the first approximation of the optimum temperature to maximize the non-dimensional power when the piston speed is not zero and when therefore both internal and external irreversibilities are accounted for.

Furthermore, the next step in the approximation procedure is to replace *T_x_* in Equation (74) by Equation (78), that allows obtaining a more accurate expression of Φ term:

(79) $$\Phi_{w}^{\prime }={\left[1+C\left(1-\sqrt[{4}]{\frac{T_{0}\Phi_{w}}{T_{H,S}}}\right)\right]}^{-1}.$$

One could continue the iteration, but the gain in accuracy would become insignificant. Thus, the optimized temperature of the cycle fluid at the hot end of the engine coming out of TFS analysis is:

(80) $$T_{opt}^{{}^{\prime }\left(w\neq 0\right)}=\sqrt{\frac{T_{H,S\cdot }T_{0}}{\Phi_{w}^{\prime }}},$$

and the maximum non dimensional power output of the internally and externally irreversible Carnot cycle becomes:

(81) $$P_{ND,\max Z}=\left(1-\frac{T_{opt}^{{}^{\prime }\left(w\neq 0\right)}}{T_{H,S}}\right)\left(1-\frac{T_{0}}{\Phi_{w}^{\prime }T_{opt}^{{}^{\prime }\left(w\neq 0\right)}}\right)={\left(1-\sqrt{\frac{T_{0}}{T_{H,S}\Phi_{w}^{\prime }}}\right)}^{2}.$$

Then, the efficiency of the irreversible Carnot cycle is calculated by substituting $T_{opt}^{{}^{\prime }\left(w\neq 0\right)}$ into Equation (67) that leads to:

(82) $$\eta_{act}=1-\sqrt{\frac{T_{0}}{T_{H,S}}\Phi_{w}^{\prime }}\cdot \left[1+C\left(1-\sqrt[{4}]{\frac{T_{0}}{T_{H,S}}\Phi_{w}^{\prime }}\right)\right].$$

One can see now that Equation (82) unifies the FTT and TFS analyses by the same expression of the actual efficiency of an irreversible Carnot cycle engine. Thus:

- For internally reversible, externally irreversible Carnot cycle engine for which *w* = 0 and consequently, $\Phi_{w}^{\prime }$ = 1, one gets the Curzon–Ahlborn “nice radical” [3]:

(83) $$\eta_{CA}=1-\sqrt{\frac{T_{0}}{T_{H,S}}}.$$

- For an internally and externally irreversible Carnot cycle engine for which *w* ≠ 0 and consequently, $\Phi_{w}^{\prime }$ > 1, one gets:

(84) $$\eta_{act}=1-\sqrt{\frac{T_{0}}{T_{H,S}}}\zeta_{w},$$

with

(85) $$\zeta_{w}=\sqrt{\Phi_{w}^{\prime }}\left[1+C\left(1-\sqrt[{4}]{\frac{T_{0}}{T_{H,S}}\Phi_{w}^{\prime }}\right)\right].$$

Note that $\zeta_{w}\geq 1$ and it accounts for internal irreversibilities of the cycle when depending on the piston speed. Equations (83)–(85) clearly show that the nice radical of FTT analysis overestimates the actual efficiency of the engine evaluated by TFS analysis.

A second unification attempt is under development. It aims to extend the modeling by considering, in addition to the finite speed, two other causes of internal irreversibility: friction and throttling.

Based on previous equations of the first unification attempt, a new expression was derived for the actual efficiency of the Carnot cycle engine:

(86) $$\eta_{act}^{irr}=1-\frac{T_{0}}{T_{X}}\left\{1+4\left(\frac{aw}{c_{1}}+\frac{\Delta p_{f}}{p_{av,34}}+\frac{\Delta p_{thr}}{p_{av,34}}\right)\frac{\left(1-\sqrt{\frac{T_{0}}{T_{X}}}\right)}{\left(\gamma -1\right)ln\frac{p_{2}}{p_{3}}}\right\},$$

where Δ*p_thr_* is estimated as [62,63,64,83]:

(87) $$\Delta p_{thr}=C_{thr}\cdot w^{2},$$

with *C_thr_* = 0.005.

Then, the irreversibility coefficient yields:

(88) $$C_{irr}=4\left(\frac{aw}{c_{1}}+\frac{\Delta p_{f}}{p_{av,34}}+\frac{\Delta p_{thr}}{p_{av,34}}\right)\frac{1}{\left(\gamma -1\right)ln\frac{p_{2}}{p_{3}}}.$$

The power output and efficiency of the Carnot cycle engine with finite speed processes considering all internal irreversibility causes are smaller compared to those determined from Equations (81) and (82), since the new correction is more substantial by its three terms (Equation (88)). 

The results of this modeling emphasize optimum speed values generating maximum power output, as well as the effect of irreversibilities on the optimum cycle high temperature.

## 3. Results

**Figure 4:**
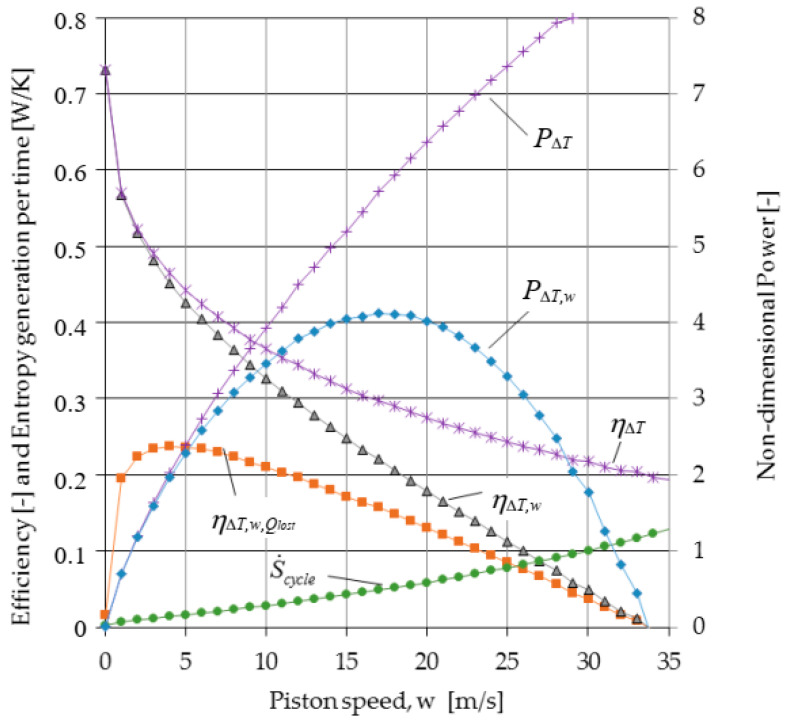
Power, efficiency and entropy generation per time as a function of the piston speed.

**Figure 5:**
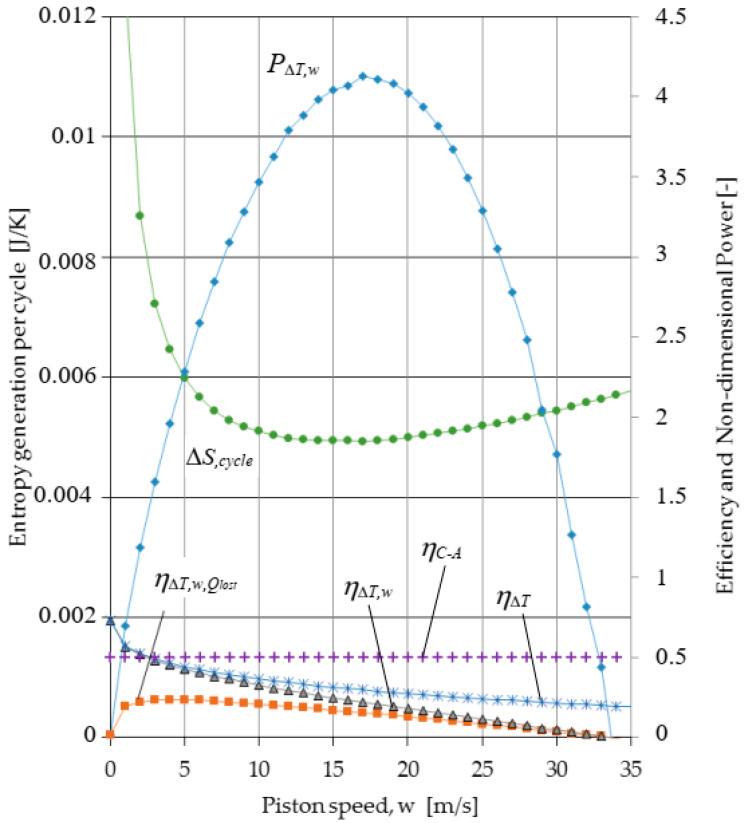
Power, efficiencies and entropy generation per cycle as a function of average piston speed.

**Figure 6:**
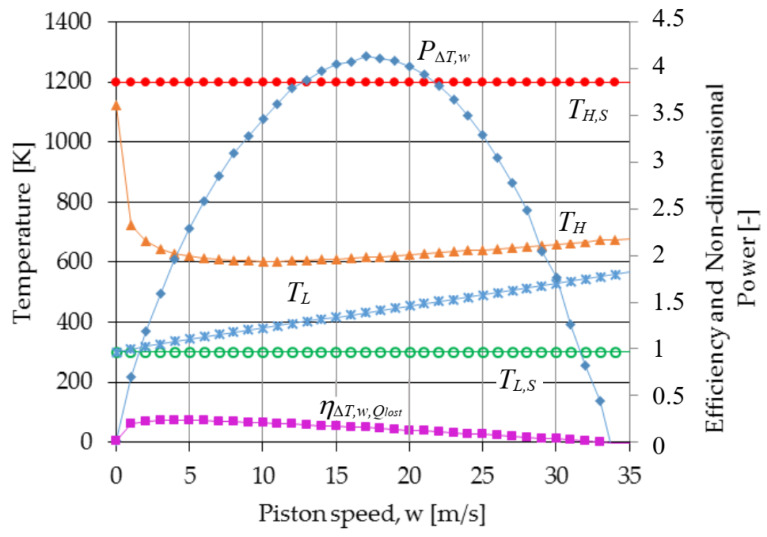
Power, efficiencies and temperatures as a function of average piston speed.

The results of TFS analysis presented in Section 2.2 relative to a Carnot cycle engine with internal and external irreversibilities generated by losses due to (1) heat transfer between the cycle and the heat source and sink, (2) the effect of variation in the area for heat transfer and in the dwell time for heat transfer due to the movement of the piston during the isothermal expansion and compression processes, and (3) non adiabaticity of the engine are presented in Figure 4, Figure 5 and Figure 6. The following fixed parameters entering in the equations of the model were used: *D* = 0.015 m; *L*_1_ = 2 m; *ε* = 3; *f* = 0; *p*_1r_ = 0.05 bar (pressure of the gas in state 1r); Δ*p_f_* = (0.97 + 0.045 w)/80; *T_H,S_* = 1200 K; *T_L,S_* = 300 K; *γ* = 1.4; *B_ins_* = 0.002 m; *k_ins_* = 0.01 W/mK; *D_e_* = 0.019 m. The cycle fluid is air that is considered as an ideal gas with specific heat, conductivity, and viscosity varying as a function of temperature.

Figure 4 illustrates the effect of irreversibilities introduced gradually on the power output showing the important difference between the cycle power output for the reversible Carnot cycle and for the Carnot cycle with irreversiblities due to the finite speed of the piston. Then, the cycle efficiency including internal and external irreversibilities, $\eta_{\Delta T,w,Q_{lost}}$, is represented as a function of piston speed showing optimum values for maximum performance. In addition, the time rate of entropy generation is added in order to compare the optimization results in terms of optimal speed.

One can see that the piston speed for maximum efficiency is only 4 m/s, for which the rate of entropy generation (per unit of time) is very low. Moreover, the piston speed for maximum power is near 17 m/s, and the rate of entropy generation (per unit of time) at this speed is significantly higher. As expected, the power output decreases, as additional irreversibilities are included in the analysis.

Figure 5 brings together the efficiency of the Carnot cycle determined by the TFS analysis when it is gradually affected by irreversibility, the one based on Curzon–Ahlborn analysis, the power output, and the entropy variation per cycle as functions of piston speed. The efficiency of the Carnot cycle as determined by TFS analysis is at all piston speeds less than the efficiency based on the Curzon–Ahlborn analysis. In addition, for piston speeds greater than *w_opt_*, the efficiency of the Carnot cycle at maximum power as determined by TFS is less than the efficiency based on the Curzon–Ahlborn analysis, even if only the external irreversibility is included. For example, the TFS efficiency, at the speed corresponding to maximum power, is 0.29 when only external irreversibilities are included and is 0.15 when both internal and external irreversibilities are included in the analysis.

An important aspect is related to the entropy generation per cycle and per time as functions of piston speed from Figure 4 and Figure 5. Their evolution with the piston speed is completely different, in that only Δ*S_cycle_* shows a minimum for the speed as the maximum power output.

The hot and cold heat reservoir temperatures, the hot and cold end gas temperatures, and the Curzon–Ahlborn optimized temperature are shown in Figure 6 as a function of the piston speed. The hot-end gas temperature optimized for maximum power is shown to be nearly the same over a large variation range of piston speeds (5 to 10 m/s), as the Curzon–Ahlborn optimized temperature. In addition, the predicted temperature difference between the high and low gas temperature is shown to increase as the piston speed decreases and to be especially great at piston speeds less than the speed for maximum efficiency. 

**Figure 7:**
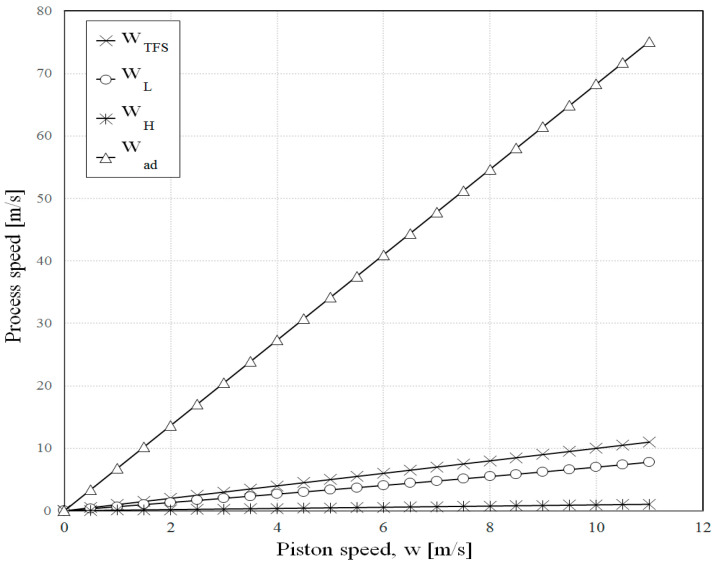
Piston speeds for process in TFS and FTT analyses as function of average piston speed.

**Figure 8:**
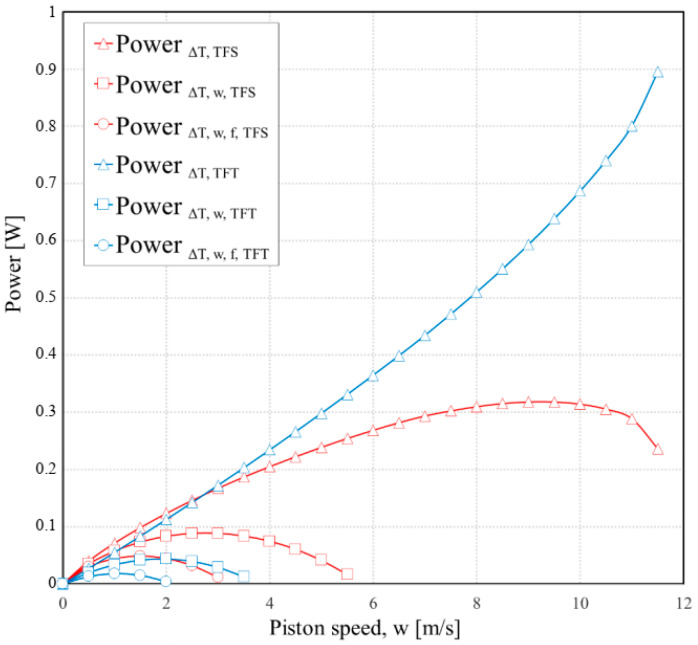
Power output of the Carnot engine for processes in TFS and FTT optimizations as function of average piston.

**Figure 9:**
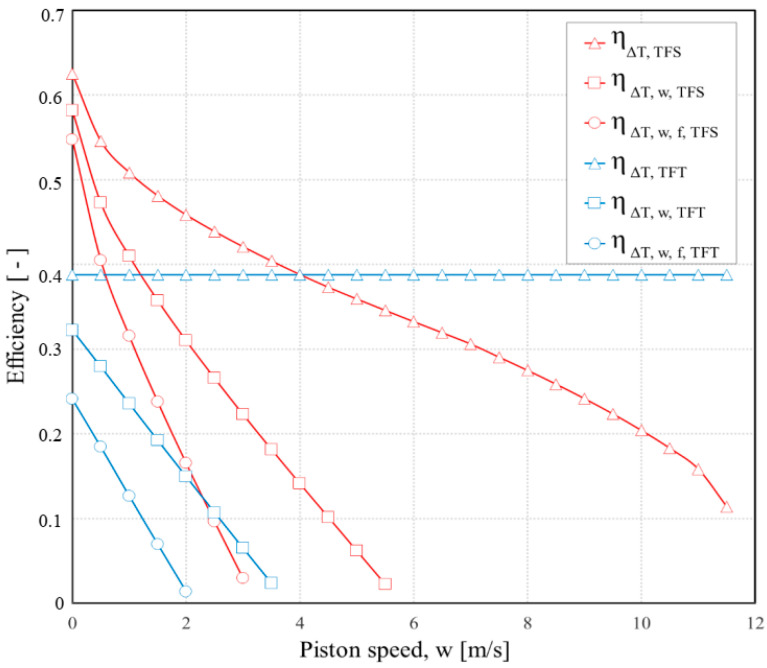
Carnot cycle efficiency based on TFS and FTT optimizations as function of average piston speed.

Some results of the second model (Section 2.3) are shown in Figure 7, Figure 8 and Figure 9.

Figure 7 illustrates the relative speed of the adiabatic processes and of each of the isothermal processes in FTT optimization compared to the average speed of the piston considered in TFS optimization. The curves show that the optimization results in lower speed than the average speed of the piston *w_TFS_*, for the two isothermal processes in FTT optimization. In addition, the high temperature isothermal process has the lowest speed; then, it follows the low temperature isothermal process with a higher speed, while the adiabatic processes occur at a much higher speed. However, the internal irreversibilities were not included in the original Curzon–Ahlborn analysis [3], so the high piston speed during the adiabatic process had no negative effect on the cycle efficiency and power. In fact, the resulting slower piston speed during the isothermal processes significantly enhanced the cycle efficiency and power in FTT optimization.

The effect of the piston speed on the power output and efficiency for a Carnot engine with external irreversibilities and internal ones gradually introduced in both TFS and FTT analyses is shown in Figure 8 and Figure 9, respectively. These results are based on the following fixed parameters: *D* = 0.015 m; *L*_1_ = 0.5 m; *ε* = 2; *f* = 0.3; *a′* = 1.1; *p_1r_* = 0.01 bar (pressure of the gas in state 1_r_); Δ*p_f_* = (0.97 + 0.045 w)/60 bar; *T_HS_* = 800 K; *T_LS_* = 300 K; *γ* = 1.4.

The FTT optimization predicts greater power output from the Carnot engine at almost all piston speeds than the TFS optimization when only external irreversibilities (Δ*T*) are considered. It is due to the little cycle time that was allocated to the adiabatic processes in the FTT optimization. This allowed more time for the isothermal processes without any penalty associated with the more rapid adiabatic processes, since the internal irreversibilities of these processes are not considered. In the TFS optimization for example, at 9 m/s the power is 0.33 W, and the efficiency is 25%. In the FTT optimization at the same speed, by comparison, the power is 0.6 W, and the efficiency is 39%. However, when the internal irreversibilities are included in the analyses, the TFS optimization results in greater power and efficiency than FTT, even though both are less than when the internal irreversibilities were neglected.

It is also important to keep in mind that a cycle that operates with three different piston speeds for the four processes presents a huge mechanical complication in the design of the actual engine. While it may be possible to design such an engine (for example, using cams with different profiles for each process), there is no need to do so, since the TFS optimization predicts superior operating performance.

**Figure 10:**
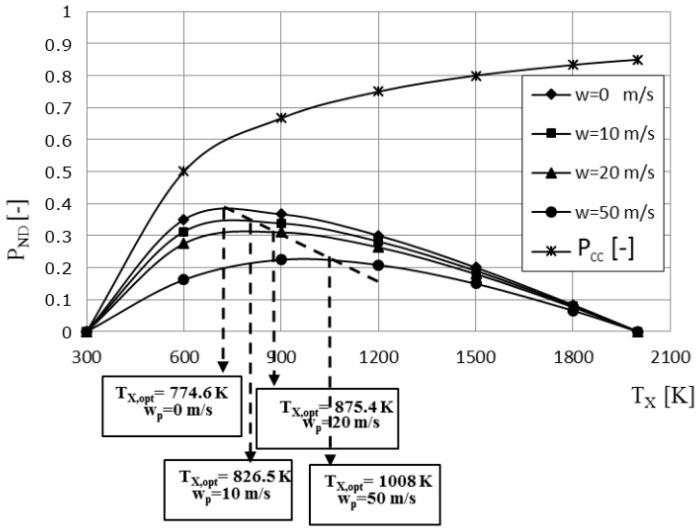
The non-dimensional power of the Carnot cycle engine as a function of the cycle high temperature and the piston speed *w*, as determined from TFS analysis.

The non-dimensional power as determined from Equation (77) as a function of the cycle high temperature and the piston speed is shown in Figure 10. In addition, the power output of the reversible Carnot cycle is added for comparison purposes. The non-dimensional power reveals the maximum value for any fixed piston speed or internal irreversibility consequence, and this maximum is moving toward growing temperature *T_x_* as the piston speed increases.

**Figure 11:**
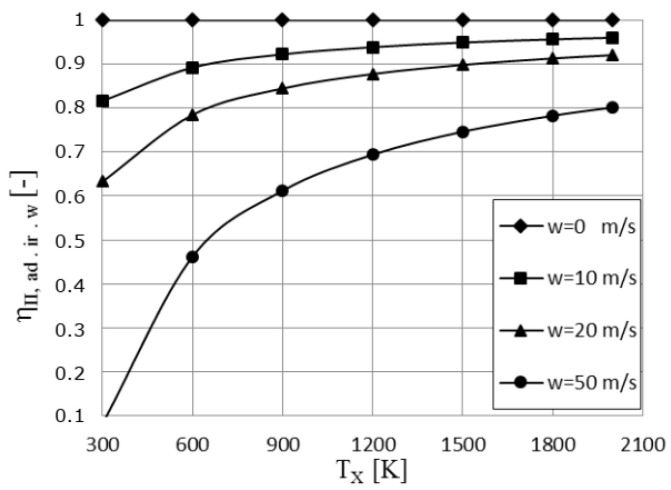
The effect of the piston speed, *w*, on the second law efficiency variation with the cycle high temperature.

Figure 11 presents the second law efficiency variation versus the cycle high temperature for different values of the piston speed. The curves show that this irreversibility coefficient decreases as piston speed increases, as expected, and the decrease is more important at lower values of the cycle high temperature.

**Figure 12:**
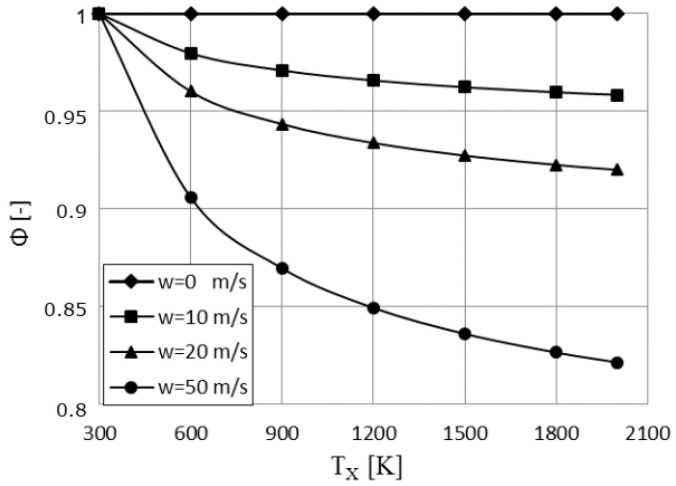
Parameter Φ variation with the cycle high temperature *T_X_* for different piston speed values.

Regarding the irreversible term Φ determined from Equation (74), its variation with the cycle high temperature and piston speed becomes important mainly at high speeds, as illustrated in Figure 12. However, there is little change of Φ in the region of optimal temperatures (from 800 to 1000 K).

**Figure 13:**
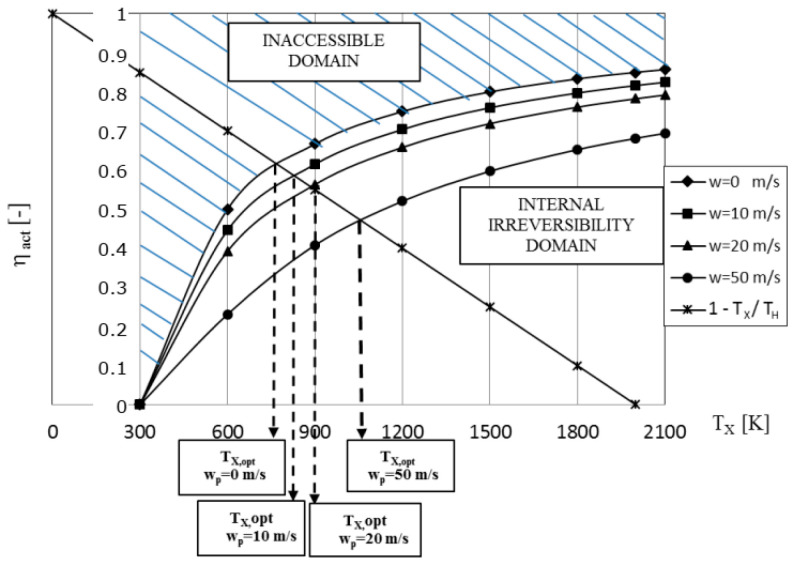
Graphical determination of optimal temperature.

The comparison of the results before (Figure 10) and after (Figure 13) using approximations in search of optimal temperature expression that optimizes the power output of the engine shows good agreement and lends confidence that a first iteration provides sufficiently accurate results for most purposes. However, it is possible to improve the accuracy of the results by making a new iteration.

## 4. Conclusions

Important performance parameters of an irreversible Carnot cycle engine based on optimization models developed in Thermodynamics with Finite Speed and by using the Direct Method have been presented. This analysis predicts lower values of Carnot cycle efficiency than is predicted by the Thermodynamics in Finite Time (FTT), as originated by Chambadal and Curzon–Ahlborn. The piston speed for maximum power and for maximum efficiency has been found for two sets of engine parameters, and it has been shown that entropy generation per time clearly differs from entropy generation per cycle. Moreover, a minimum occurs for the entropy generation per cycle at optimum piston speed corresponding to maximum power.

This study produces a more realistic model for the design of Carnot cycle engines since it includes many of the various internal and external irreversible processes that occur in the actual operation of these engines and correlates them with the finite speed of the piston. 

The present analysis has shown that the first unification attempt of TFS and FTT optimization involves analytical correction of the Curzon–Ahlborn efficiency, which is well known as a nice radical, by a term accounting for internal irreversibilities of the Carnot cycle engine. They were evaluated based on the Fundamental Equation of TFS, the First Law for Processes with Finite Speed, where the main irreversibility causes are accounted for, namely, finite speed of the piston, friction, and throttling. This correction appears not only in the Carnot cycle efficiency but also in the optimum temperature of the gas at the hot end of the engine for maximum power, and in the non-dimensional power output of the engine. Thus, the engine performances were derived analytically for a Carnot engine with external and internal irreversibilities generated by finite speed w.

A step further in this first unification approach did a comparison between TFS and FTT optimization results for a Carnot cycle emphasizing that TFS analysis can account for both kind of irrevesibilities, and it can also provide improvement of FTT results.

Thermodynamic analysis based on the Direct Method and Finite Speed of the processes is shown to be especially effective for engineering optimizations since the efficiency and power can each be optimized based on gas temperatures and process speed. The fact that it is already used by other researchers [54,55,56,57,58,84,85,86,87] proves its capability to become a useful tool in thermal machine analysis and optimization.

We do hope that this work marks an important step toward the development of a more powerful Engineering Irreversible Thermodynamics, which could be a synthesis unifying the three important branches, namely Thermodynamics with Finite Speed, Thermodynamics with Finite Dimensions, and Thermodynamics in Finite Time.

## Data Availability

The data presented in this study are available on request from the corresponding author.
